# Mercury and artisanal and small-scale gold mining: Review of global use estimates and considerations for promoting mercury-free alternatives

**DOI:** 10.1007/s13280-023-01843-2

**Published:** 2023-03-10

**Authors:** Susan Keane, Ludovic Bernaudat, Kenneth J. Davis, Malgorzata Stylo, Nellia Mutemeri, Patience Singo, Pontsho Twala, Itai Mutemeri, Anne Nakafeero, Imelda Dossou Etui

**Affiliations:** 1 NRDC, 1152 15th St, NW, Suite 300, Washington, DC 20005 USA; 2 UN Environment Programme, 8-14 Avenue de la Paix, 1211 Geneva 10, Switzerland; 3 University of Witwatersrand, Private Bag 3, WITS, Johannesburg, 2050 South Africa; 4 No 1, Patel Close, Kiwafu, Kansanga, Kampala Uganda; 5 School of Mining Engineering, University of Witwatersrand, Private Bag X3, Johannesburg, 2050 South Africa; 6 135, Melrose Arch, Johannesburg, 2076 South Africa; 7 NEMA House, Plot 17, 19 & 21, Jinja Road, P. O. Box 22255, Kampala, Uganda

**Keywords:** Artisanal and small-scale mining, ASGM, Gold mining, Mercury, Minamata Convention

## Abstract

Artisanal and small-scale gold mining (ASGM) is present in over 80 countries, employing about 15 million miners and serving as source of livelihood for millions more. The sector is estimated to be the largest emitter of mercury globally. The Minamata Convention on Mercury seeks to reduce and, where feasible, eliminate mercury use in the ASGM. However, the total quantity of mercury used in ASGM globally is still highly uncertain, and the adoption of mercury-free technologies has been limited. This paper presents an overview of new data, derived from Minamata ASGM National Action Plan submissions, that can contribute to refining estimates of mercury use in ASGM, and then assesses technologies that can support the phase out mercury use in ASGM while increasing gold recovery. The paper concludes with a discussion of social and economic barriers to adoption of these technologies, illustrated by a case study from Uganda.

## Introduction

Artisanal and small-scale gold mining (ASGM) is a widespread activity, present in over 80 countries and producing up to 20% of the world’s gold. It employs approximately about 15 million miners, of which an estimated 4–5 million are women and children, and is the source of livelihood for 100 million people, often in rural and remote areas where few economic alternatives exist (UNEP 2019a). Further, due to the recent increase of global gold prices, ASGM is expected to be on the rise (Yoshimura et al. 2021).

Because of the high price of gold and the minimal steps required to create the final bullion, ASGM should be an ideal vehicle for transfer of wealth to poor, rural mining communities, resulting in poverty reduction. However, complicated legal systems, lack of institutional support, lack of awareness of appropriate technologies and long value chains (often on the margin of legality) mean that many mining communities remain in poverty, and the sector continues to use mercury as the primary mechanism of gold extraction.

For artisanal and small-scale gold miners, mercury amalgamation provides a simple and inexpensive solution to recover gold. The process entails combining mercury with gold-bearing ore or concentrate. A gold-mercury amalgam is formed, which is heated to vaporize mercury, leaving behind the gold. Mercury is widely available, even in remote communities, and it enables miners to produce gold (and thus income) on a daily basis. This means that mercury amalgamation is often the default solution, and as a result, ASGM remains the largest user and emitter of mercury globally.

Given these complex circumstances, the Minamata Convention on Mercury, a global treaty aimed at reducing mercury pollution, created a separate article (Article 7) to address mercury use in ASGM (UNEP 2019b), which has heightened attention to the issue. However, although the ASGM sector has been addressed in development projects for many years, the full extent of ASGM operations and the magnitude of mercury use, and mercury emissions to air and releases to land and water, remain uncertain. Further, the adoption of mercury-free technologies is limited, mainly at a few sites which have benefitted from international assistance. Replication and generalization of interventions have been hampered by miners’ lack of knowledge on geological context and more appropriate processing techniques, and by a lack of access to finance for the ASGM communities (planetGOLD Programme 2020).

This paper presents information on the estimated magnitude of mercury use in ASGM and the potential for its replacement. The section "Baseline data on mercury use in ASGM" describes previous estimates of mercury use in the global ASGM sector and highlights the prospects for extracting new data from Minamata ASGM National Action Plans, which are submitted by Parties to the Minamata Convention. The section "Existing and emerging methods for non-mercury ASGM processing technologies and workflows" describes technologies that can be deployed to replace mercury use, while the sections "Lessons from global efforts on promoting mercury-free technology" and "Social and economic considerations for adoption of mercury-free technologies: a case study of Uganda" highlight the social and economic challenges to the adoption of these technologies in real-world conditions, using Uganda as a case study.

## Baseline data on mercury use in ASGM

### Global Mercury Assessment 2018

The most recent published global estimates of mercury use in ASGM by country appear in the 2018 Global Mercury Assessment (hereafter referred to as the 2018 GMA) (UNEP 2019a). Because of the typical informality of the sector, and thus lack of official data regarding its operations, the 2018 GMA estimates drew on diverse data sources including trade publications, donor reports, conference materials, peer-reviewed literature, and personal communications with miners, traders, and government officials. The year of the most recent data available ranged from 1992 to 2017 for the 80 countries where ASGM was reasonably estimated to be present. Recognizing the large and differing uncertainties, each country estimate includes an error range, from 30 to 100%, depending on the data quality.

According to the 2018 GMA, the total annual volume of mercury used in ASGM (that is, the amount applied to gold ore minus the amount recovered and recycled) was estimated to be 2059 tonnes (with a range from 897 to 3131 tonnes). Annual global mercury emissions to air from this use were estimated at 838 tonnes (with a range from 675 to 1000 tonnes). This represents about 38 percent of total annual estimated anthropogenic air emissions from all sectors. The large majority of ASGM mercury use was found to occur in countries in South America, Sub-Saharan Africa, and East and Southeast Asia. The 2018 GMA estimates of mercury emissions from ASGM were about 23 percent higher than those in the 2013 Global Mercury Assessment (UNEP 2013). Regionally, emissions increased in South America and Sub-Saharan Africa during this period, while decreasing in Asia (primarily due to a large decrease in estimated emissions from China). However, it is likely that these apparent trends reflect increased information on ASGM in many countries. It is unknown how much of the difference reflects real increases in mercury use.

The 2018 GMA estimates are a useful starting point in quantifying ASGM mercury use, but large uncertainties as well as lack of recent data sources in some countries limit the value to policymakers and researchers. In addition, the 2018 GMA only provided estimates of mercury use and emissions and did not report on other dimensions such as total gold produced from ASGM number of miners, worst practices, and spatial distribution of sites. The next section investigates how data from Minamata ASGM National Action Plans can help fill these gaps.

### Minamata ASGM National Action Plans

#### Background on the NAP process

**Fig. 1:**
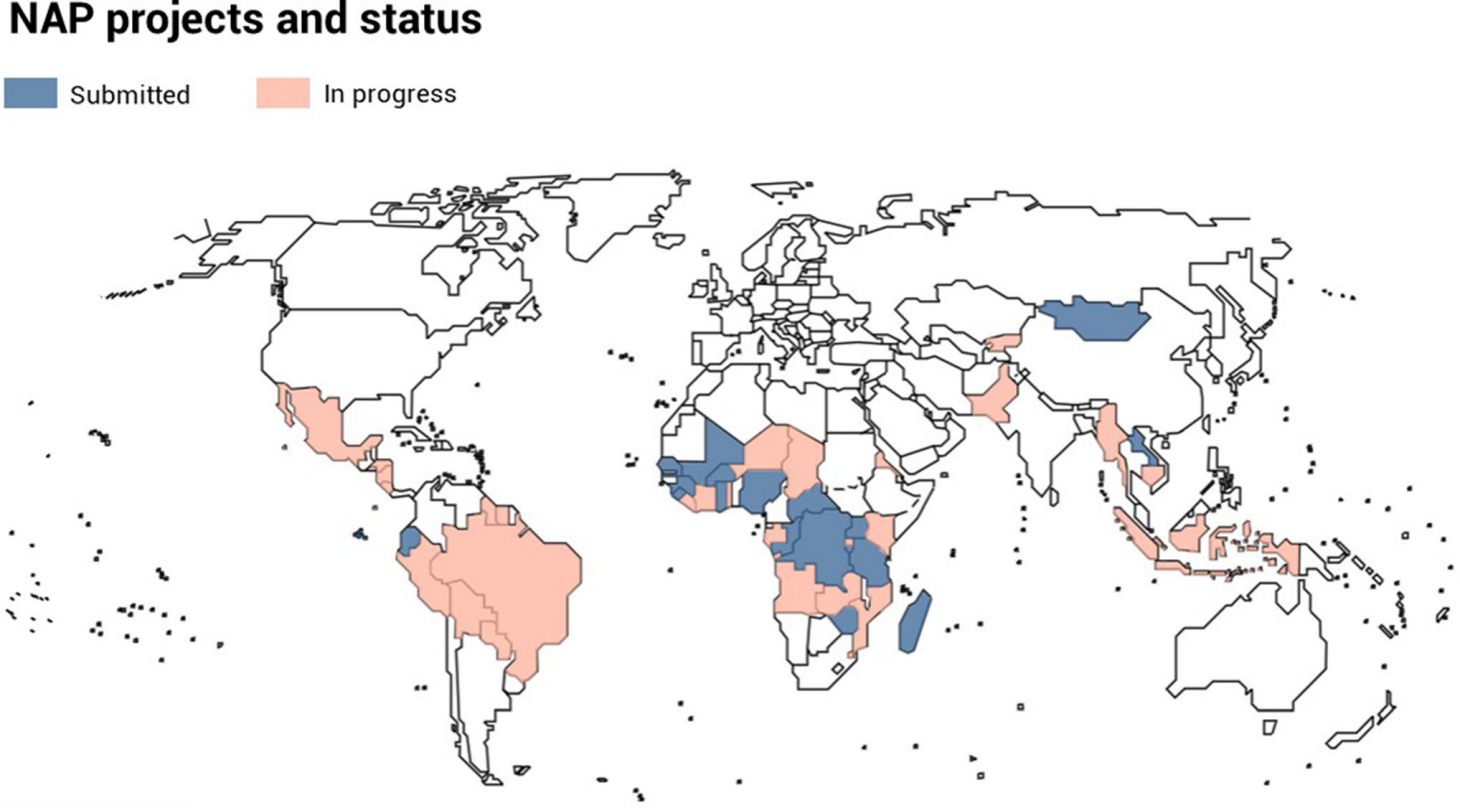
National Action Plan projects and submission status

Article 7 of the Minamata Convention on Mercury requires the development of a National Action Plan (NAP) to reduce, and where feasible eliminate, the use of mercury in ASGM for each Party that determines that ASGM in its territory is “more than insignificant”. To date, 46 countries have started NAP projects (Fig. 1). NAP development typically takes two to three years and is usually financed by the Global Environment Facility (GEF). A Party is required to submit their NAP to the Secretariat of Minamata Convention no later than three years after entry into force of the Convention for the Party or three years after notifying the secretariat that ASGM activity is “more than insignificant” in a country, whichever is later (UNEP 2019b). Out of 46 countries that have undertaken NAP projects as of this writing, 18 have finalized and submitted their NAP documents to the Secretariat of Minamata Convention.^1^

The development of a NAP requires a good understanding of the ASGM sector to support formulation of realistic and effective strategies and to measure progress in implementation. To gain this understanding, countries first collect data on mercury use and practices employed from ASGM sites in the field and through stakeholder consultations and literature review, as well as socio-economic, health and environmental information. Countries are encouraged to use O’Neill and Telmer (2017) as a common guidance in this process. Since the NAP projects are relatively recent (the first NAP was submitted in 2018 with many projects still ongoing) these data typically reflect the most recent information on the sector.

#### Research approach for NAP data

To assess the wealth of information generated in the NAPs, quantitative and qualitative data has been extracted from the eighteen final NAP documents submitted to the Secretariat of the Minamata Convention (represented in blue in Fig. 1). The quantitative data categories include: (i) estimated amount of mercury used by the sector, (ii) estimated amount of gold produced by the sector, (iii) estimated number of miners (where applicable gender disaggregated) involved in the sector, (iv) and mercury reduction targets. In addition, qualitative information has been gathered, including presence of worst practices, as well as various environmental, health, and socio-economic information documented in the NAPs. Finally, the reported NAP strategies for each country were summarized and added to the database. The extracted data and information were analyzed, and interactive dashboards were created to facilitate data exploration.^2^ It should be noted that the eighteen NAPs analyzed are not a representative global sample of ASGM countries but are those which have been submitted at the time of writing.

#### Preliminary analysis of NAP data

##### Comparing mercury estimates

**Fig. 2:**
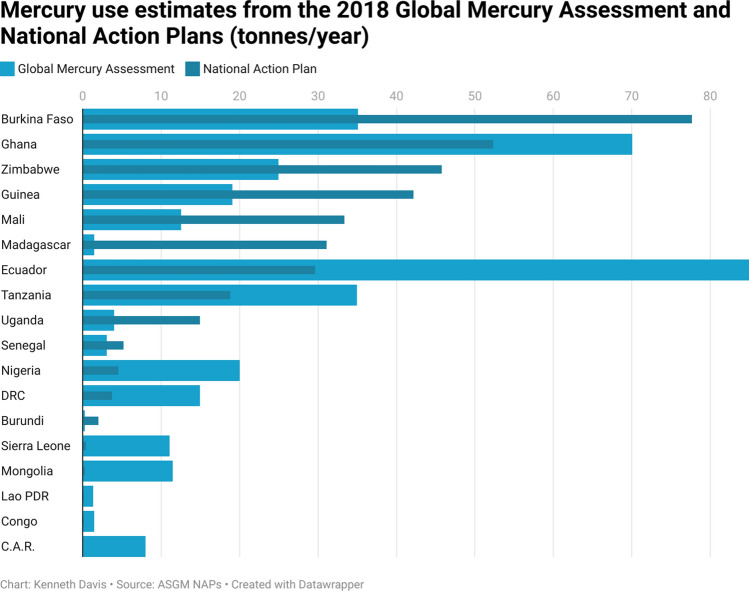
Mercury use estimates from 2018 Global Mercury Assessment and National Action Plans (tonnes per year). NAP mercury use estimates from Sierra Leone, Mongolia, Lao PDR, Congo and Central African Republic (C.A.R.) are all under 0.35 tonnes/year

The estimated quantities of mercury used in ASGM per country, as reported in NAPs, were compared with the previous estimates reported in the 2018 GMA (Fig. 2). In about half of the cases, NAP data indicates a significant increase in the estimated amount of mercury use in contrast to previous estimates (UNEP 2019a). For example, in the case of Madagascar and Burundi, estimates increased by over tenfold. In other cases, for example in Ecuador, Democratic Republic of the Congo (DRC) or Mongolia, the NAP estimates are lower than previously reported amounts. These differences do not necessarily mean that mercury use changed during this time, but likely reflect, in part, changes in the availability of information collected through the nationwide field studies undertaken by the NAP projects and might reflect the different methodologies used for the data collection. For example, the Madagascar NAP documented several previously unknown, mercury-intensive small-scale dredging operations, resulting in a significant increase in mercury use above previous estimates (including the 2018 GMA). Conversely, Ecuador reported in their NAP that only 40% of the ASGM gold is produced using mercury. This finding resulted in a notably lower mercury use estimate than in the 2018 GMA.

##### Presence of worst practices

**Fig. 3:**
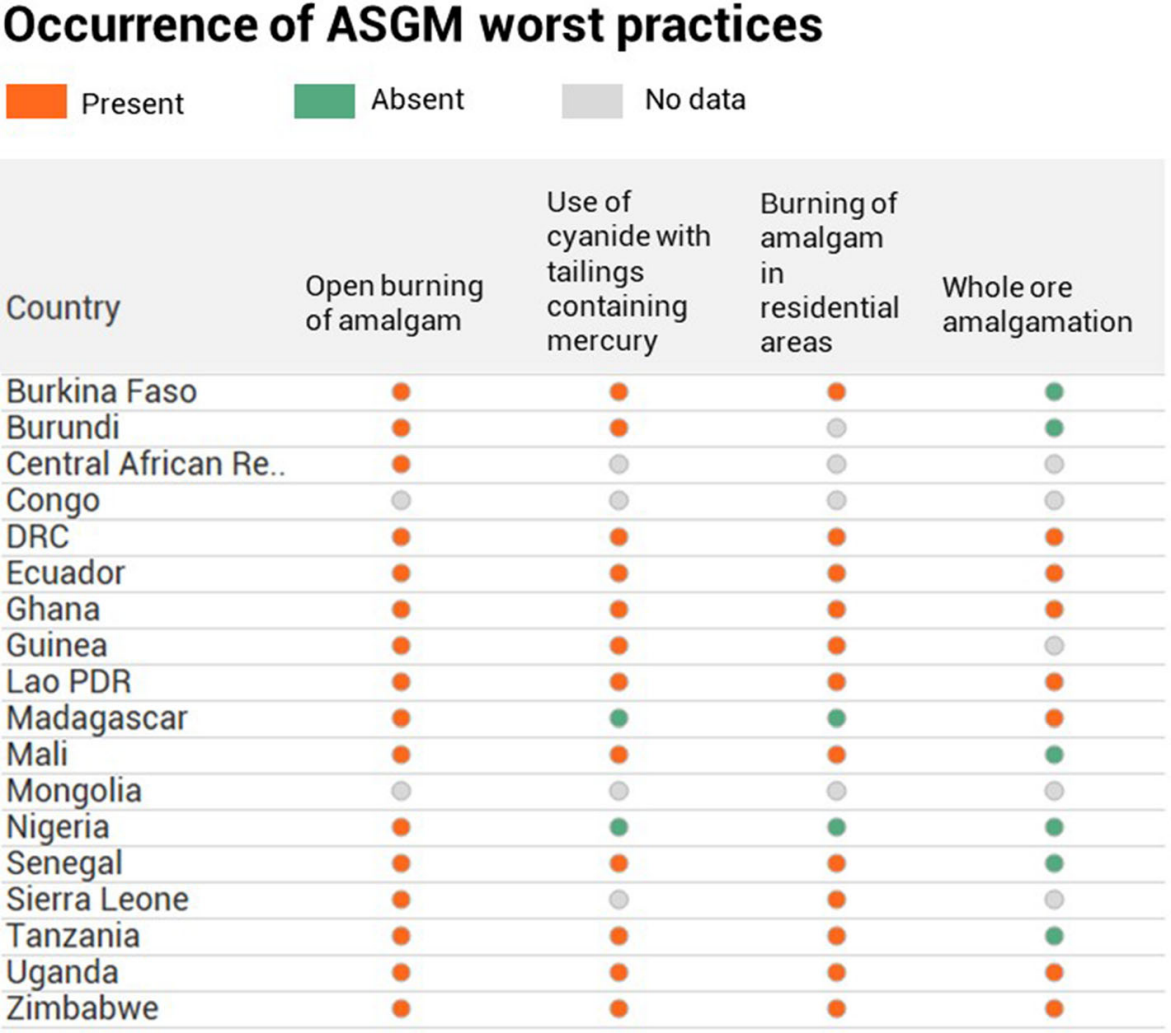
Occurrence of ASGM Worst Practices as reported in National Action Plans

The Minamata Convention specifies that each NAP elaborate strategies to eliminate four “worst” practices, defined as (i) whole ore amalgamation, (ii) open burning of amalgam (i.e., without a mercury capture device), (iii) burning of amalgam in residential areas, and (iv) use of cyanide on mercury containing tailings or sediment without first removing mercury. Country reporting on these practices is shown in Fig. 3. The most common worst practice, as reported by 14 out of 18 submitted NAPs, is open burning of amalgam. Most of the countries also reported burning of amalgam in residential areas which puts the miners, their families, and the surrounding communities in danger of direct exposure to mercury vapor. Cyanide leaching of tailings or sediment containing mercury has been reported in 12 out of 18 countries. Out of remaining six countries, two (Madagascar and Nigeria) explicitly reported absence of this practice, while the other four countries did not provide information. The least common worst practice is whole ore amalgamation. Its presence was reported in 6 out of 18 countries. Burkina Faso, Burundi, Mali, Nigeria, Tanzania, and Senegal explicitly reported absence of this practice within their territories.

##### Mercury use versus gold production

**Fig. 4:**
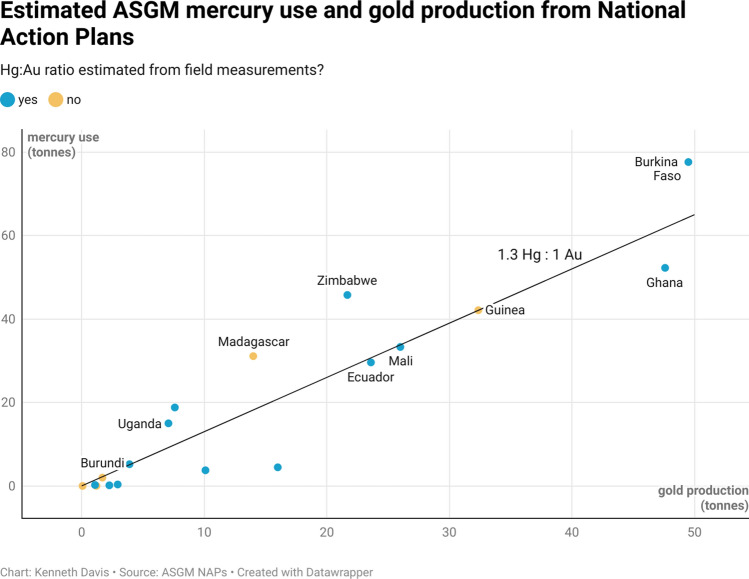
Estimated mercury use versus gold production from National Action Plans

Approximately 20% of the world’s gold is produced by ASGM (Seccatore et al. 2014; Yoshimura et al. 2021). Based on the submitted NAPs, 278 tons of ASGM gold is produced in 18 countries. Figure 4 plots the estimated quantities of ASGM gold production against the estimated quantities of mercury use by country. Based on 18 country data, the mercury to gold ratio (denoted as Hg:Au) is about 1.3:1. Thirteen out of 18 countries have specifically mentioned that the estimated Hg:Au ratio is based on field measurements, while the other five have used default ratios in the absence of the field measurements. Countries with more mercury intensive ASGM operations include Zimbabwe, Uganda, and Madagascar, whereas less mercury intensive operations were observed in the Democratic Republic of the Congo (DRC), Nigeria, Sierra Leone, and Guinea. While most of the countries (13 out of 18) did not report whether ASGM gold is entirely or only partially produced using mercury, five countries provide approximate percentage of ASGM gold being produced using mercury, including Ecuador (40%), Guinea (60%), Uganda (73%), Mali (88%) and Zimbabwe (96%).

##### Mercury reduction targets

**Fig. 5:**
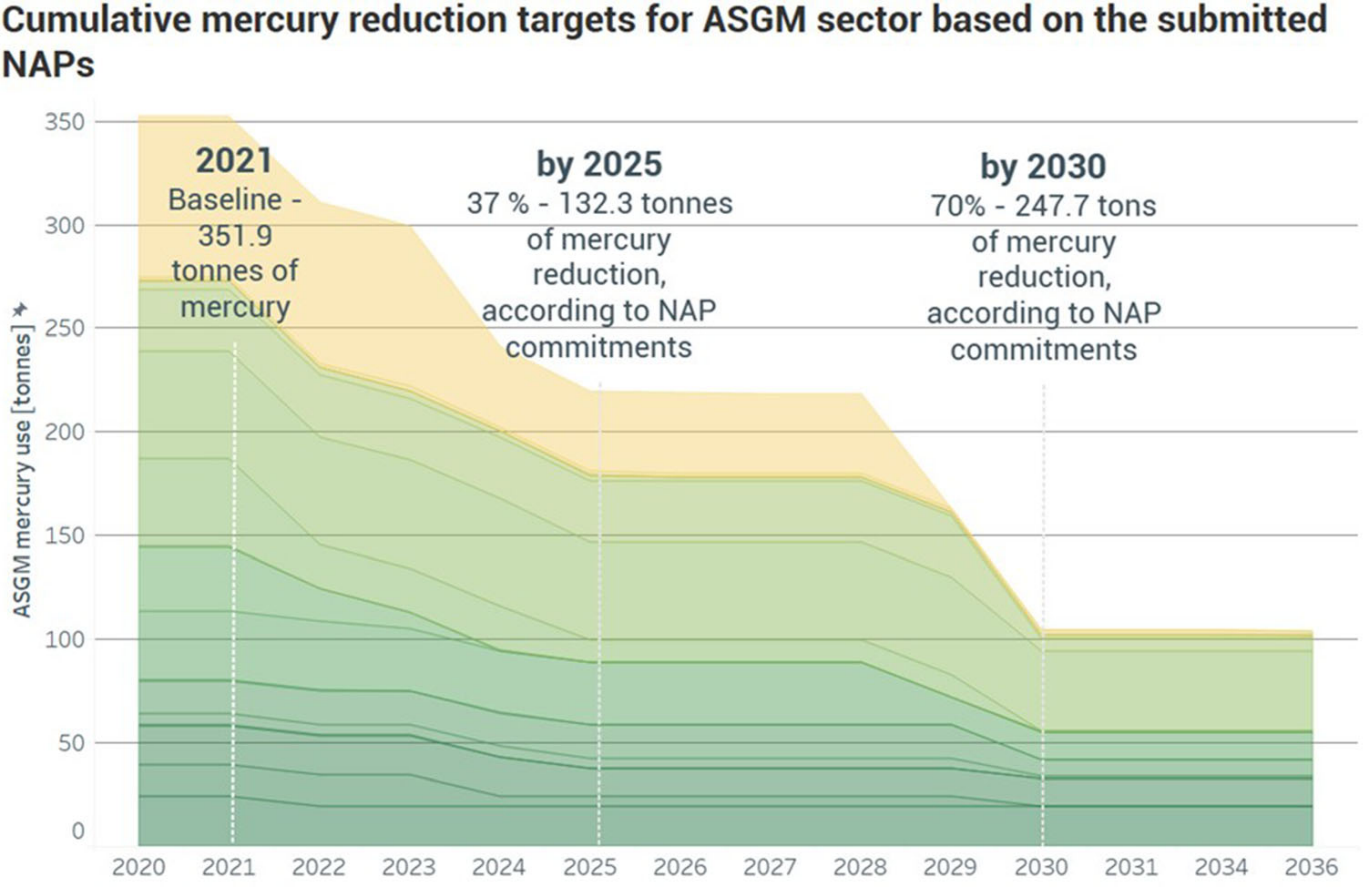
Cumulative ASGM mercury reduction targets reported in National Action Plans

Mercury reduction targets reported in the NAPs (Fig. 5) provide an indication of when and by how much these countries intend to reduce mercury use. Baseline mercury use reported by the 18 countries is 352 tonnes per year. By 2025, 37% of that amount, or 132.3 tons, is targeted for elimination by implementing actions outlined in the NAPs. By 2030, 247.7 tons of mercury, or 70% of the baseline amount, is targeted for elimination by the countries.

#### Expectations as more NAPs are finalized

More than half of the countries with reported ASGM presence in the 2018 GMA have started or finalized their NAPs (46 out of 80). As of this writing, 28 of these 46 countries have not yet submitted the document. According to the 2018 GMA, four of those 28 (Indonesia, Peru, Bolivia, and Brazil) are among of the most mercury-use intensive ASGM countries (Table 1).

**Table 1:** NAP status in the top ten most mercury intensive ASGM countries

Country	Average Hg use [t/y] per Global Mercury Assessment 2018	Minamata convention status	NAP development status
Indonesia	427.0	Party	Ongoing
Peru	327.0	Party	Ongoing
Colombia	175.0	Party	No project
Bolivia	120.0	Party	Ongoing
Brazil	105.0	Party	Ongoing
Venezuela	102.0	Party	No project
China	100.0	Party	No project
Ecuador	85.0	Party	Finalized
Sudan	83.0	Non-Party	No project
Philippines	70.0	Party	No project

These top ten most mercury-use intensive ASGM countries account for an estimated 1594 tons of mercury use annually, or around 77% of the global total (UNEP 2019a). However, until now, among these top ten countries, only Ecuador has submitted its NAP to the Secretariat of Minamata Convention. The availability of additional data as the new NAP documents are submitted will provide further insights into global ASGM dynamics and compliment the preliminary analysis demonstrated in this paper by providing the most recent quantitative figures and qualitative information surrounding ASGM sector. The 34 countries where ASGM presence was identified (UNEP 2019a) that have not undertaken a NAP project account for nearly 25% of global mercury use annually in ASGM (UNEP 2019a), underlining the need for further NAP project development and implementation to characterize the remaining mercury use in ASGM around the world.

### Gaps, challenges and lessons learned in collecting ASGM mercury use data

Challenges with data collection for NAPs are largely related to the informality of the ASGM sector (O’Neill and Telmer 2017; UNEP 2019c). In most, if not all, of the countries that have developed their NAPs, high levels of informality, and in some countries, legal prohibitions on the use of mercury in ASGM, inhibit open dialogue with miners, which is a significant obstacle to collection of reliable data on key aspects such as mercury supply and use, gold production, trade dynamics and flows, and gender dimensions. For example, only the Lao People's Democratic Republic was able to provide a quantitative estimate of mercury traded per year. Amongst the African countries, some (e.g., Burkina Faso, Burundi, DRC, Guinea, Madagascar, Mali, Nigeria, Senegal, Sierra Leone) reported illegal/informal inflows from neighboring countries; others, such as Congo, did not provide any information on mercury trade mainly because of the secrecy surrounding its use. Zimbabwe was able to identify, with some precision, the points of entry of mercury into its territory. In Latin America, Ecuador mentioned black-market import of mercury from Mexico via Peru and Bolivia but did not provide any quantitative estimates.

Another gap relates to the lack of detailed geographical information on ASGM sites in many NAPs. Most NAPs mention the main regions, provinces, or districts where ASGM occurs, and many also include maps depicting these areas. But few attempt a detailed and comprehensive geographic inventory of sites. Admittedly this is a difficult task, due to the sheer number of sites, their inaccessibility, and limited time and resources for field data collection.

Various sources of information and diverse methodologies might also amplify the observed differences between data sets. For example, although all NAPs have a common guidance (O’Neill and Telmer 2017), a number of different techniques for site level analysis and extrapolation to the national level are used for the NAP baseline assessments. Further, the many other sources of ASGM information at the national and local level, including academic studies (e.g., Wilson et al. 2015; Yoshimura et al. 2021) and civil society initiatives, represent a wide range of data quality and variable methods, which contributes to lack of comparability.

Finally, most baseline estimates fail to quantify uncertainty, including through more rigorous methods such as error propagation or Monte Carlo analysis, which could help researchers and policymakers better understand the state of knowledge of the sector (Schwartz et al. 2023). Instead, uncertainties are often provided in a qualitative manner listing challenges and gaps linked to the presented quantitative data.

As a result of these challenges and uncertainties, collecting reliable data and developing national overviews of the ASGM sector in NAPs has proven to be a complex task that requires specific capacity-building, considering local realities. Lessons learned from ongoing and completed projects will help to strengthen and harmonize existing approaches to obtaining representative and comparable data at local, regional, and international levels. Many countries have expressed the need for additional capacity-building on the use of tools for collection and interpretation of data and baseline estimates (UNEP 2019c). These trainings, which could be extended to local practitioners in the gold mining sector, could facilitate better access to sites and strengthen communication with ASGM actors, but also guide investigations and define precise site selection criteria to be representative of the national context.

## Existing and emerging methods for non-mercury ASGM processing technologies and workflows

### Evaluating alternatives to amalgamation

To reduce the use of mercury in the ASGM sector, government- and donor-funded interventions over the past decades have promoted technology solutions aimed at providing alternatives to the amalgamation process. According to the World Bank (2020), the first mercury elimination programs targeting ASGM were implemented in the 1980s and 1990s. Since then, there has been a growing inventory of mercury-free technologies identified and promoted; however, their adoption in the ASGM sector remains low. This section provides an overview of the landscape of mercury-free gold processing technologies,^3^ then assesses the technologies’ suitability for the ASGM sector, and finally identifies main operational shortcomings that need to be addressed to improve adoption.

### Factors influencing suitability of mercury-free gold processing technologies

**Fig. 6:**
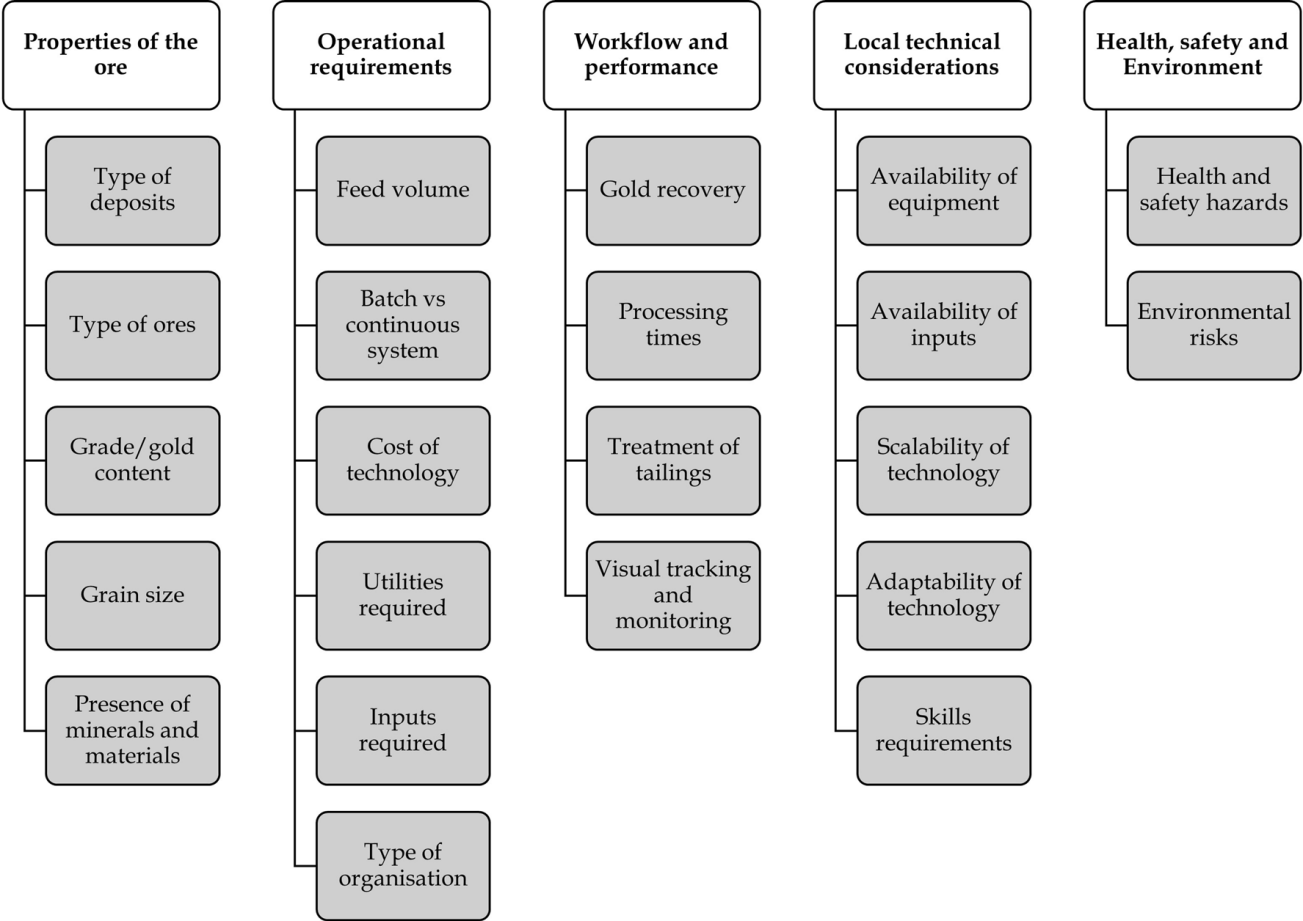
Key factors for assessing suitability of mercury-free gold processing technologies

Assessing the suitability of mercury-free technologies for use in ASGM is challenging because of the complex and varied environments in which the sector’s activities take place. Figure 6 presents various factors that can be used to determine such suitability, including the properties of the ore, operational requirements, workflow and performance of the technologies, local technical considerations, and health, safety, and environmental aspects. For mercury-free technologies to compete with the amalgamation process, they need to be applicable to different types of gold ore typically processed by ASGM operators; they must be suitable for small and large batches of ore; and they should offer high gold recoveries at less time compared to the amalgamation process (Esdaile and Chalker 2018). Because ASGM activities are conducted in operating environments characterized by highly varied production levels and technical knowledge of the miners, it is also important that the technologies incorporate local needs and capacities (Hinton et al. 2003).

Hinton et al. (2003) note that the likelihood of technologies being adopted in ASGM depends on their simplicity compared to the amalgamation process. The availability of equipment and material (chemicals, steel, rods, piping, generators, etc.) also influences the level of adoption and use of the technologies.^.^ Esdaile and Chalker (2018) also note that the technologies and inputs (i.e., reagents) need to be safe, easy to handle and generate biodegradable waste, if possible. Social, economic, cultural, legal and governance issues also have a bearing on the adoption of mercury-free technologies, as covered in sections "Lessons from global efforts on promoting mercury-free technology" and "Social and economic considerations for adoption of mercury-free technologies: a case study of Uganda" below.

### Research approach for assessing technologies

Table 2 describes the framework used in this research to assess suitability of the existing and emerging mercury-free technologies to ASGM.

**Table 2:** Framework for assessing mercury-free gold processing technologies

Assessment category	Overarching questions
Properties of the ore	Are the technologies suitable for the different properties of ore being treated in the ASGM sector?
Operational requirements	Are the combined costs of the technologies affordable to the different categories of the miners/operations in ASGM?
Workflow and performance	How do technologies compare to the amalgamation process in terms of processing workflow, gold recovery rates, and ability to recover other minerals?
Local technical considerations	Do the technologies suit local needs in terms of the availability of the technology, required supplies and inputs, scalability, and adaptability requirements?
Health, safety, and environment	Does the recovery of gold using the individual technologies pose any health, safety, and environmental risks?

A total of 48 technologies were identified, based on a review of technical papers, peer-reviewed journals, reports/updates, and grey literature. After compiling the list of technologies, targeted searches were conducted on the different technologies related to the technical characteristics noted in Fig. 6. The data collected regarding these characteristics was then evaluated against the questions in Table 2. Notably, while a number of sources were identified that describe these technologies, there were few published field reports or studies uncovered in publicly available literature that describe the detailed performance of these technologies under conditions typical of ASGM operations.

### Results and analysis

#### Catalogue and landscape of the technologies

**Fig. 7:**
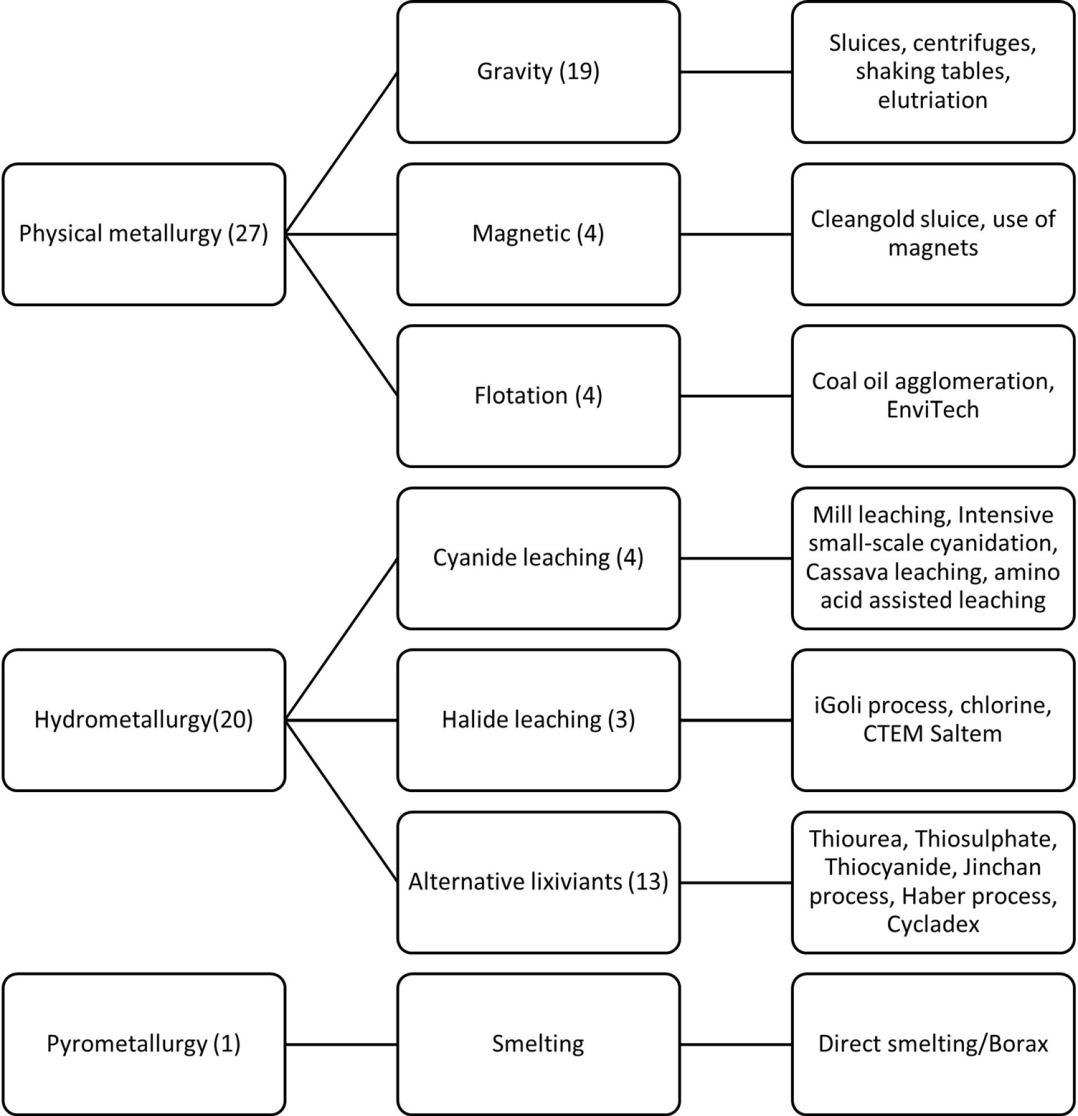
Categorization of mercury-free gold processing technologies

Figure 7 categorizes the identified technologies into the three broad areas of extractive metallurgy: physical metallurgy, hydrometallurgy, and pyrometallurgy (Rosenqvist 2004). Physical metallurgy uses ore properties such as shape, size, density, color, magnetic susceptibility, and others to initiate separation of minerals (Poloko 2019). All hydrometallurgy processes are chemically based and differences in the extraction methods are in the type of lixiviants used (e.g., cyanide, halides/chlorine, thiourea, thiosulphate and others). Lastly, pyrometallurgy methods include smelting, roasting, refining and calcination (Poloko 2019).

Of the 48 technologies that were identified, most fall under physical metallurgy (*n* = 27) with gravity-based methods being in the majority (*n* = 19). The other technologies use magnetic (*n* = 4) and flotation methods (*n* = 4) to recover gold from the ore material. Examples of physical separation-based technologies include centrifugal concentrators (e.g., Gold Kacha, iCON concentrator, Knelson concentrator, Knudsen bowl, Falcon concentrator, Blue Bowl concentrator), shaking tables (e.g., Gemeni shaking table, Holman-Wilfley shaking table), sluices (Cleangold sluice, GoldMasta) and elutriation (e.g., Goldrop). Centrifugal concentrators consist of a rotating bowl which is fitted with grooves and riffles. As the bowl receives the feed, centrifugal force is generated and this results in the heavy minerals (e.g., gold) settling on the grooves/riffles. The separation of light and heavy minerals is often assisted by the injection of pressured water which maintains the fluidization of the heavy particles on the grooves. The shaking tables and sluices depend on the flow of water on a slope and supporting surfaces to separate the heavy gold particles. The Cleangold sluice, for example, consists of polymetric magnetic sheets inserted in an aluminum sluice.

The other technologies that were identified include jigging and coal–oil agglomeration. Jigging uses alternating water pressure to separate heavier and lighter materials, recovering coarse gold (usually nuggets) from oversized ore material (Appropriate Process Technologies 2022). Coal–oil agglomeration involves the recovery of free gold particles in a slurry containing coal–oil agglomerates which have been created by intensive agitation of a mixture of coal and oil in water. The method takes advantage of the hydrophobicity of coal and gold compared to other material in the ore (Otsuku and Yue 2017).

Under hydrometallurgy, 20 technologies were identified. Of these, four technologies use cyanide to leach the gold from the ore. Another three technologies use halide-based reagents, and the majority are based on alternative lixiviants. The use of cyanidation to recover the gold is increasing in ASGM and as such, it has been observed that responsible cyanidation must be part of the solution (Stapper et al. 2021). However, it must be noted that the Minamata Convention on Mercury has identified the specific practice of cyanidation of tailings contaminated with mercury as a "worst practice” that should be eliminated due to the creation of highly toxic and bioavailable mercury-cyanide complexes (Lennett and Gutierrez 2014).

Two emerging cyanide-based leaching processes were identified: cassava leaching, and amino acid assisted leaching. Cassava leaching is an experimental technology that uses liquid cyanide extracted from cassava as a substitute to commercial cyanide. According to Torkaman et al. (2021), cassava and other cyanogenic plants as sources of cyanide offer cost savings as well as environmental benefits because the cassava liquid is waste produced from the production of flour. Amino acid assisted leaching uses a mixture of cyanide and glycine to recover gold from the ore. Glycine is an amino acid that bonds well with minerals that are difficult to separate from gold during the cyanidation process. For example, glycine bonds well with copper, leaving the cyanide to leach gold from gold-copper concentrates (Minerals and Process Solutions 2023).

Halides are used as the lixiviant in systems such as the iGoli mercury-free gold extraction system, CETEM Saltem process, and chlorine leaching. The iGoli technology uses a mixture of hydrochloric acid and sodium hypochlorite to dissolve gold in a concentrate; the gold in the solution is then precipitated using sodium metabisulphite (Mahlatsi and Guest 2003). Some technologies use lixiviants such as thiourea, thiocyanate, and thiosulphate (planetGOLD Programme 2021). Some companies promote proprietary lixiviants, such as Cycladex (Cycladex 2023), Jinchan Gold Dressing Agent (Beyuo et al. 2016) and CNLITE ECO Leaching Agent (CNLITE 2023).

The last category is pyrometallurgy. Direct smelting is a traditional method of producing gold doré from clean concentrates by using heat to separate gold from impurities that are present in the concentrate. Smelting can be done with several fluxes; however, borax (sodium tetraborate) is the most commonly used.

#### Assessment of the technologies

##### Properties of the ore

Most of the technologies identified can work with different ore deposits under at least some circumstances. However, effectiveness of gravity concentration is limited by mineralogy, gold liberation and gold particle size. Alluvial deposits generally consist of free milling ores which are mostly liberated. Most of the technologies work with this type of ore because of the ease of extraction. Hard rock deposits may contain free milling ore and refractory ores. Free milling ores in hard rock deposits can be liberated through crushing and grinding. Refractory ores are more challenging because gold particles are occluded in other minerals making liberation difficult (De Michelis et al. 2010).

Of the technologies identified, only a few were found to be capable of recovering gold from refractory ores, including coal–oil agglomeration and some leaching technologies. Calvez et al. (1998) indicated that the coal–oil agglomeration process can recover gold from sulfide ores if sulfides are less than 5 percent of the feed. Grayson (2007) noted that thiosulphate is capable of leaching gold from refractory ores [in particular, carbonaceous ores and carlin-type ores (these are sediment-hosted gold bearing ores)].

Other key ore characteristics are gold grain size and grade. Some of the technologies can recover fine and coarse gold in the same ore feed (the coal-agglomeration process) and a few technologies can process both low and high-grade material. Some technologies like iGoli require high-grade feed, that is more than 25 percent gold. Notably, most of the technologies have been designed for small batches, and few are suitable for larger processing facilities (Veiga et al. 2014). There are, however, a few technologies have different models that can cater to artisanal operations as well as those with higher production outputs (such as the Knelson concentrator, iCON concentrator, HPC Technology (EXTRAC-TEC 2023), and Gemeni shaking table).

##### Operational requirements and costs

The technologies evaluated vary widely in cost and operating requirements, Costs can be as low as USD 40 for a prospector’s sluice to USD 45 000 or more for an integrated plant with various equipment such as the Mercury-Free Alluvial Technology set up by Commodity Monitor in Ghana (Commodity Monitor 2021). Few of the technologies are found to be affordable to artisanal-scale miners, and some of the technologies may be beyond the means of small-scale mining operations that are not well capitalized.

Affordability assessments should also consider the costs of utilities and other inputs. Most of the technologies use water, with most gravity-based technologies requiring clean and consistent water supply. Also, most of the technologies require electricity.

##### Workflow and performance

Most of the mercury-free technologies have the potential to outperform the amalgamation process with respect to gold recoveries, although this depends on ore type. For example, Torkaman and Veiga (2022) reported gold recoveries of approximately 84% with cyanide leaching versus approximately 19% with mercury amalgamation, in a study on Colombian gold ore. The potential for higher recovery and thus increased profits can be a key motivator for adoption of new technology (although other factors, discussed later, can limit adoption despite this potential). Centrifuges and shaking tables improve the efficacy of gravity concentration circuits, with highly efficient shaking tables found to be effective for recovering gold as fine as 32 μm (UNEP 2012). Some technologies are also able to recover other minerals that may be present in the ore such as silver, platinum etc. While some technologies can produce smeltable gold directly, most of the technologies need to be used together with other technologies (i.e., either upstream or downstream). This adds to the costs and complexity of the workflow and necessitates technical training and skills.

##### Local technical considerations

The uptake of mercury-free technologies depends on their local availability, and their inputs, scalability, and adaptability at the local level. Most of the technologies are sourced through global agents, though replicas may be available locally. According to Veiga (2004), about four Brazilian manufacturers produce copies of Knelson Concentrators at about 10% of the cost of the original. Adaptability as assessed is linked to local availability of inputs like electricity and water as well as ease of transportation to remote areas. Some technologies come as mobile units that can be transported for use in remote areas.

##### Health, safety, and environmental aspects

One benefit of many of the technologies, when well-managed, is lower health, safety, and environmental risks compared to mercury use. The reduction in environmental impacts is linked to the use of less/non-toxic or more biodegradable reagents, the recyclability of the lixiviants, introduction of tailings/effluents neutralizing reagents and the ability to collect and re-use process water. The only potential environmental risks noted for most of the gravity-based technologies are the consumption of large amounts of water and tailings generated during processing, but the use of water and generation of tailings are also concerns for existing mercury-based processes.

### Summary

Based on the technical characteristics discussed above, mercury-free technologies exist that have the potential to improve the recovery of gold by:

- Improving initial concentration to provide feed to the next stage of concentrate upgrading and final gold extraction.
- More efficiently upgrading concentrates and thus replacing mercury in the final stage of gold extraction.
- Producing smeltable gold concentrate.
- Providing new cyanide-based methods that reduce toxicity and impacts on the environment.
- Offering alternative lixiviants that can replace both mercury amalgamation and cyanidation.

As noted earlier, while there is extensive literature describing these mercury-free technologies, there is limited information on field performance and uptake of the individual technologies. The few published field reports also tend to be very site-specific, making generalization to other types of sites difficult. Another major data gap relates to how very specific mineralogical characteristics of ores relate to the performance of the technologies, limiting the ability to evaluate the suitability of mercury-free technologies in these circumstances.

## Lessons from global efforts on promoting mercury-free technology

### Introduction and research approach on technology adoption experiences

Since the late 1970s, ASGM has been recognized as a driver of rural development, resulting in significant changes in approaches to its governance (Hruschka 2011) and to the introduction of alternative technologies (Priester 1993). Since that time, dissemination of mercury-free technology has remained a key global strategy to eliminate mercury use in ASGM. Yet to date, adoption is limited.

To understand factors affecting adoption of these technologies, published and grey literature sources were reviewed. Interventions to promote the technologies were categorized into application of *traditional or mature technologies* versus application of *emerging technologies*. The *traditional technologies* are those that are mature and have been commercialized in the mainstream gold processing sector and ASGM. These have been used in large-scale gold mining with a proven track record of performance under appropriate conditions (Lehmann 2020). The *emerging technologies* are those that have been mainly implemented at a laboratory scale, with few trials under ASGM conditions in the field. Some of them are established in industrial mining applications.

The literature review yielded little publicly available peer-reviewed documentation on the efficiency, challenges, and gaps related to promotion of either traditional or emerging technologies in ASGM settings. The evidence that does exist focuses on traditional technologies, including sluices, jigs, spirals, centrifuges, shaking tables, cyanidation, and direct smelting (Veiga et al. 2018). Therefore, to complement the literature review, information on practical field experiences to introduce mercury-free technology was collected through consultations with key informants, including academics, ASGM practitioners, mining engineers, and technology providers working in ASGM. Fifteen consultations were conducted with ASGM experts and practitioners involved in mercury-free initiatives in Ecuador, Honduras, Ghana, Guyana, Indonesia, Mongolia, Peru, Philippines, Sudan, Suriname, Uganda, and Zimbabwe. The field experiences were evaluated with regard to:

- Maturity of the technology employed,
- Perceived technical performance,
- Successful approaches to promote these technologies, and
- Challenges faced in the interventions.

### Results

This research revealed that challenges to adoption fall into six categories: (1) technical aspects, (2) miner’s level of organization, (3) the development process of the technology, (4) technology complexity, (5) degree of supply chain collaboration, and (6) responsiveness to miners’ needs.

#### Technical aspects

Although the performance of technologies depends on ore characteristics (Vieira 2001; Veiga et al. 2006; Drace et al. 2021), limited understanding of mineralogy of ASGM ores means mercury-free technology deployment is often a “one size fits all” circuit, with predictably mixed results. The production of high-grade concentrates for direct smelting requires rich, free milling ores, which may lead to high-grading (also known commonly as cherry-picking) resulting in poor resource utilization. Further, some local downscaled versions of technologies are poorly optimized for mineralogical characteristics, leading to inefficient gold recovery conditions (Wills and Finch 2015). Interventions may fail to recognize that technology needs change as ore characteristics change in an operation. For example, mines transitioning from oxidized free milling ores to refractory sulfide-rich zones will need to adjust circuits to maintain high recovery (Presad et al. 1991).

#### Level of organization of miners

Technology adoption also depends on how miners are organized. ASGM is characterized by low throughput and small gold volumes recovered by individuals or small groups. Mercury-free technologies may not match the needs and scale of miners' production. Direct smelting has encountered major obstacles amongst miners recovering very small amounts of gold due to perceived potential gold losses during the smelting process. In Uganda, miners intimated that the use of expensive and large shaking tables failed because miners had a small production capacity that did not match the large feed required; miners opted for amalgamation instead (P. Andesu, Tira Landlords Small Scale Miners Association, personal communication; P. Obbo, Busia United Small Scale Miners Association, personal communication). In contrast, some technologies have been successfully adopted by organized and high-capacity ASGM, tolerant of longer processing times. (Swiss Agency for Development and Cooperation 2013). In particular, cyanidation has gained popularity as a mercury alternative in Peru, Ecuador, Uganda, Sudan, and elsewhere, amongst leach plant operators and ASGM groups with financial and management capacity (Hruschka 2011). Despite the potential effectiveness of cyanide leaching as a mercury-free solution, proliferation of cyanidation plants poses environmental challenges with contaminated tailings discharged into rivers such as Calera and Amarillo in Ecuador (Velasquez-Lopez et al. 2010).

#### Development process of technology

Successful adoption of technology in an ASGM setting is linked to its evolution through various development stages, including laboratory and field testing, optimization to mineralogical conditions and user feedback. Emerging ASGM processing technologies, including small scale flotation, thiosulphate leaching, use of cassava for leaching, coal–oil agglomeration, amino acid leaching and elutriation (Hilson and Monhemius 2006; SGS 2008; Torkaman et al. 2021) have been tested at laboratory or pilot scale but have not gone through the technology development cycle from concept, laboratory testing, piloting, upscaling, and commercialization (Logsdon et al. 1999), nor are there adequate field reports on pilot actions (Lehmann 2020). Some researchers have expressed concern that technologies promoted and commercialized without adequate evidence of field efficacy will fail to be viable commercially (Marsden and House 1992).

#### Complexity of technologies

Challenges faced by emerging technologies include the requirement for specialized expertise, expensive reagents, supply of clean water, larger ore volumes, longer processing times and increased regulatory requirements. These requirements limit their application to highly organized ASGM and undermine wider adoption (Hilson and Monhemius 2006).

#### Degree of supply chain collaboration

Some interventions encourage mercury-free gold processing through collaboration along the supply chain, rather than dissemination of technology directly to miners (Veiga and Fadina 2020). In these collaborative efforts, miners sell ores or gold-bearing tailings directly to mercury-free (generally cyanide) processing plants and/or mining companies. Veiga and Fadina (2020) report that co-existence of conventional mining companies and ASGM is successfully gaining momentum. Through the collaboration, miners focus on mining while processing companies use mercury-free approaches, eliminating mercury across the value chain.

The collaborative approach also has potential to increase miners' incomes because of improved gold recoveries associated with cyanidation. One model developed in Ecuador has additional features that also benefit miners. In the model, miners are given access to accredited and reliable assay laboratories at a 50% discount; waiting times at processing centers are reduced, as miners only deliver ore for assaying rather than wait for processing; and miners are immediately paid 20% of the value of their ore based on assay values (PAGE and Ministry of Energy and Non-Renewable Natural Resources 2021). In Mongolia, the legal framework requires miners to use approved mercury-free centralized processing centers, but these may not provide the most efficient gold recovery circuits, so the significant gold remains in the tailings. In co-existence arrangements, the tailings from these plants are sold to approved large scale cyanide leaching operators, thus preventing the clandestine treatment of these tailings with mercury for further gold recovery (Swiss Agency for Development and Cooperation 2013; A. Bayarsaikhan, planetGOLD Mongolia, personal communication). In the Philippines, the large scale mine Acupan supports ASGM by allowing miners to operate some sections of the mine area and supporting them with equipment and explosives. The ASGM operators, in turn, integrate their ore production to the Acupan circuit and are paid based on assay values (Benguet Corporation 2005).

Although these collaborations form for commercial and compliance-related reasons, they still result in mercury use reduction. However, the co-existence model does not work well in all cases, as some processing centers take economic advantage of miners, reducing the economic benefits (Mukono et al. 2018; Veiga 2020). A close analysis of terms of these collaborations needs to be undertaken to ensure fairness for the parties involved.

#### Responsiveness to the needs of the miners

ASGM communities and local experts should be involved in mercury-free gold interventions from conception to implementation to achieve local ownership and gain traction for new technologies. Efforts using the “fix and go” approach, without integrating ASGM’s cultural, social, economic, and technical context (Hruschka 2011), have resulted in abandoned investments and equipment graveyards. In contrast, success in implementing mercury-free technology can be in part attributed to ASGM involvement during technology development and roll-out, and provision with appropriate expertise on the ground (Swiss Agency for Development and Cooperation 2013). The uptake of mercury-free technology can also be derailed by ASGM supply chain actor business interests, such as mercury suppliers (Spiegel et al. 2018). In addition, processing plant owners who benefit from inefficient initial processing that leaves a significant amount of gold left behind in the tailings have no interest in improving gold recovery in the initial process (Mukono et al. 2018; Veiga 2020).

Finally, personal preferences of miners also play a major role in acceptance of technology. For example, gravity concentration tests in Mongolia showed that a centrifuge recovered 5 percent more fine gold than a sluice. However, despite superior performance of the centrifuge, miners preferred the sluice because it was cheaper and easier to operate and maintain while still achieving their mercury-free gold processing objectives. With a mercury use ban, the miners’ priority was achieving mercury-free production, rather than higher recoveries (Swiss Agency for Development and Cooperation 2017). They also opted for a more expensive shaking table as it provided a solution for mercury elimination, despite losses in lower grade material. Years later, the miners prefer to shift to more sophisticated processing circuits through high recovery systems (A. Bayarsaikhan, planetGOLD Mongolia, personal communication).

### Summary of lessons learned

Several key factors that influence the uptake of mercury-free technologies include:

- Miners must be convinced they can equal or improve profits by using the technologies.
- Most ASGM operators require ongoing assistance and capacity building to be able to set up and operate new technology.
- Entrenched business models can limit the transition to mercury-free processing. These include mercury suppliers, processing plants (e.g., toll mills), and leach plant operators. The role of these actors must be considered.

When interventions fail to align proposed technologies with miners’ ore type, scale of production, nature and size of organization, and other social and economic factors, well-intentioned interventions are unlikely to succeed.

## Social and economic considerations for adoption of mercury-free technologies: a case study of Uganda

### Background

The Uganda Minamata Initial Assessment (MIA) of 2018, carried out by the Uganda National Environment Management Authority (NEMA) (NEMA 2018), estimated that ASGM in Uganda used 18 tonnes of mercury per year and accounted for 60% of total annual mercury input to the environment. These values were derived using mercury inventory toolkits (UNEP 2019d, e) designed to provide high-level mercury use estimates. Subsequent, more extensive studies (NEMA 2019) conducted as part of the NAP development process amended the estimate of annual mercury use in ASGM to 15 tonnes per year (NEMA 2019); this value was derived using methods developed by O’Neill and Telmer (2017) and UNITAR (UNITAR 2018) specifically for evaluating mercury use in ASGM, as well as through extensive stakeholder consultations.

National efforts to phase down mercury use in ASGM have, among other topics, centered on capacity-building, technical assistance, and technology transfer in the ASGM sector. For example, as detailed in NEMA (2019), demonstration sites on use of gravity concentration methods and subsequent direct smelting using borax were put in place in at Kayonza, Kassanda District from 2019 to April 2021 by National Association of Practicing Environmentalists (NAPE). Miner exchange visits to this site involving miners in Buhweju, Namayingo and Mubende districts have been carried out to spread knowledge on safer alternatives. Similar demonstration sites were constructed by Biovision-Africa (BiVA) in collaboration with the Uganda National Association of Community and Occupational Health (UNACOH), in districts of Buhweju, Busia and Amudat. However, uptake of these technologies has been limited. For example, in Buhere Village, Buhere-Bukana Sub-County, Namayingo District, where gravity separation with direct smelting has been demonstrated, artisanal gold miners were at first enthusiastic about using the mercury-free demonstration sites, but technologies were not adopted in the long run, nor were they adopted at other nearby mining sites. Similar observations have been made at the Siyanyonja mining site in Busia District, where Environmental Women for Action in Development (EWAD) (an NGO) donated a centrifuge (Gold Kacha), as well as different kinds of retorts for capturing mercury vapor, over four years ago. NEMA’s site visits revealed that no nearby ASGM association has purchased similar equipment. The retorts are also rarely used by the beneficiary miner association.

### Social factors limiting adoption

#### Limited awareness of health impacts of mercury exposures

Many miners are unaware of the health impacts of mercury, which results in miners’ continued use of mercury technologies without safety or health measures. For instance, NEMA (2019) revealed that out of 125 interviewees in districts of Busia, Namayingo, Buhweju and Bushenyi, a total of 56 (46 percent) knew nothing at all about the health risks related to the use of mercury. This was more marked in the district of Bushenyi with 73 percent having no knowledge of these health hazards. The respondents in Buhweju (60 percent), Busia (69 percent) and Namayingo (61 percent) could mention at least two mercury-related health hazards. When asked about measures of prevention of mercury exposure, most of the respondents mentioned washing hands 73 percent (*n* = 93) and use of gloves 70 percent (*n* = 85) as way of prevention of exposure to mercury. The ASGMs argued that putting health, safety and environment standards in place requires money they cannot afford. The lack of awareness of mercury hazards by land/pit owners is a particular hurdle. Most miners and gold processors do not own the land and mine pits but are hired as workers. However, the choice to avoid mercury exposure by adopting mercury-free technologies primarily depends on land/pit owners who usually do not have time to attend mercury awareness-raising meetings.

The lack of awareness about mercury presents a legitimate health concern, since there is evidence that miners and affected communities are exposed to mercury in Uganda. For instance, Tamale et al. (2016) studied mercury levels in fish species typically consumed by from Lake Albert. The study found that bellyfat of tilapia and muscle of Nile perch accumulated mercury that exceeded FAO/WHO guideline values, suggesting that these fish species should not eaten by the vulnerable groups such as children under 17 and women of childbearing age. A study by Uganda National Association of Community Occupational Health (UNACOH) (Friday et al. 2018) revealed that blood and urine of ASG miners in districts of Mubende, Busia and Ibanda had total mercury levels ranging from 43 to 136 Hg µg L^−1^ in blood and from 58 to 105.5 Hg µg L^−1^ in urine, which exceed levels of concern (5 μg L^−1^ for blood and 7 μg L^−1^ for urine) indicated in the UNEP/World Health Organization guidance for identifying populations at risk from mercury exposure (UNEP and WHO 2008). The same study revealed various symptoms (like shaking of hands and head) associated with mercury exposure and poisoning from health assessment of 160 respondents in districts Busia, Ibanda, Mubende and Amudat.

#### Land security

ASGMs lack security of land tenure because their land is communally owned, and they do not have individual certificates of ownership. This has occasioned land grabs orchestrated by other ASGMs, mining companies or their agents. Miner evictions can take place any time. Yet most mercury-free technologies are not easily movable, lessening their appeal to landless miners.

#### Traditional gender roles

Mercury-free gold processing technologies usually require more processing time than mercury technologies. This can be challenging for female miners who must attend to house chores in addition to mining. Female miners may prefer mercury technologies which are less time consuming.

#### Low levels of professionalization among miners

ASGM operations are still informally operated and do not have business training to provide basic documents like business plans, audited books of accounts or production models that demonstrate the viability of their operations. This informality affects their ability to access credit and undermines their ability to envisage the costs and benefits of mercury-free technologies. The low levels of technical education also hamper their ability to supervise, operate and maintain equipment for mercury-free technologies.

### Economic factors limiting adoption

#### Lack of economic power

Artisanal and small-scale miners in Uganda usually work on a subsistence basis, where they are only able to meet basic daily needs. They have little or no ability to save money, nor any spare income for payment of loan or lease costs. Further, socially disadvantaged populations (e.g., Dodoth and Iks) and women, whose leadership is minimal at ASGM sites, have little economic power and need targeted support to adopt mercury-free technologies.

#### Influence of middlemen

Middlemen usually finance gold extraction and processing either in cash or by providing mercury to the miners, under the arrangement that the miners must then sell all their gold to the middlemen, making gold buyers the main source of mercury supply. For this reason, middlemen may work against the adoption of mercury-free technologies. Improving access to finance and shortening the gold supply chain may earn miners more profits and reduce on the influence of these intermediaries.

#### Mercury-free gold pricing

Unless there are buyers willing to pay a premium for mercury-free gold, the price paid for gold produced with mercury-free technologies is the same as gold where mercury is used. Mercury-free gold processing techniques can be more costly, laborious and time consuming, although in the long run mercury-free technologies generally result in more gold recovery per unit of ore and thus more money for the miners. However, the initial cost capital investment hurdle must be overcome, and the business case for miners to adopt mercury-free technologies (e.g., higher gold yields) must be clear to miners.

#### Lack of public utilities

One reason that the cost of living around the mines is relatively high is the lack of public utilities. Enterprising individuals step in and privately provide basic services like water, which is fetched off site, and generators for electricity, all at a significantly higher price compared to comparable public services in urban centres with no mining activity. For example, while a twenty-litre jerrycan of piped water costs USD 0.13 in Kampala and other urban centres, the same quantity of water costs USD 0.27 in the ASGM camps. Mercury-free technologies usually require large amounts of flowing water, which is a challenge to access where ASGM sites are far away from water sources and at high elevation. Energy requirements are also usually higher for mercury-free equipment like centrifuges and shaking tables compared to simple sluices used with mercury-based techniques.

#### Limited access to capital for investment

Mercury-free technologies, especially concentrators, require initial capital investment and have high maintenance costs, compared to mercury technologies. In Uganda, the microfinancing options available in the rural areas are costly for the miners who lack the collateral required to secure the loans. The microfinance institutions are also reluctant to lend to the miners because they are considered risky due to the uncertainty of gold yields, the high migratory nature of their lifestyle and poor record keeping of gold process yields (NEMA 2019). Culturally, the gold business is considered mythical, highly uncertain, and littered with tales of theft, death, and witchcraft—a further disincentive for the microfinance institutions to be involved. Financial institutions do not treat location and exploration licences or even mining leases as assets sufficient for collateral to acquire loans. This may be largely attributed to lack of geospatial data and high costs of prospecting leading to use of personal judgment in locating gold vein and rich ore.

#### Volume of ore production needed for mercury-free technologies

In Uganda, miners tend to work alone or in small groups customarily because it may be easier to trust and work with a limited number of people. This results in the production of small amounts of ore. However, mercury-free technologies usually require high amounts of ground ore, which may not be available especially for single miners and women.

### Technical factors limiting adoption

#### Technical skills

Artisanal and small-scale miners in Uganda have limited skills in designing, construction, operating and maintaining infrastructure and equipment for mercury-free technologies.

#### Availability of inputs

Mercury is supplied by middlemen to miners at ASGM sites hence miners do not have to travel long distances to buy mercury. By comparison, inputs to mercury-free technology inputs like borax and Liquefied Petroleum Gas (LPG) come from big cities and are cumbersome to access by miners in remote areas.

#### Quality control of inputs

Quality control for mercury-free gold processing inputs like borax is not guaranteed. Variations in grain sizes affect surface area to volume ratio and hence performance.

### Recommendations to support uptake of mercury-free technology in Uganda

To support uptake of mercury-free technology in Uganda, policies and actions could be taken to address the social, economic, and technical barriers, such as:

- Mercury-free technologies that are tailored for appropriate gold ore and grade, with the aim to limit operational costs (less water and energy consumption), maintenance costs and processing time, need to be introduced. Further, the entire mercury-free processing chain should be as portable as feasible, in case of miner re-location. To reduce installation and maintenance costs, local artisans should be trained in fabrication and maintenance.
- Education of miners can be addressed by including small-scale mining, gold processing and fabrication of tools in the curriculum of public vocational institutions; creating handbooks on mercury-free gold mining appropriate for local audiences; establishing demonstration sites for mercury-free technology; and supporting miner exchange visits to the sites.
- There is a need to strategically engage community elders and local council leaders in the adoption of mercury-free technologies, and to find regulatory or other incentives for land/pit owners to adopt these technologies.
- Miners must be supported in all aspects of formalization, including acquiring mineral licenses, financial services, and extension of public utilities to remote areas. Direct engagement between ASGMs and microfinance or other types of financial institutions is key in freeing miners from the middleman’s influence, which encourages ongoing mercury use.
- Supporting the formation of associations and building trust among miners may help them mill together and produce larger amounts of ore and concentrate, which is more suitable for mercury-free technologies.
- Capacity-building and encouragement of ASGM site leadership by women is needed to address gender imbalances.
- Artisanal and small-scale gold processors could be supported to form marketing alliances to market higher prices for gold produced from mercury-free technologies. For example, this could be supported by a cell-phone based miner-to-miner communication system to enhance information sharing among miners on mercury-free gold prices. Finally, there is need to develop and update the regulatory framework on responsibly produced gold trade, which places more responsibility on buyers to incentivize mercury-free production.

For more detailed discussions of these social and economic issues and strategies to address them, readers are referred to additional information resources on ASGM formalization (IGF 2017; UNITAR and UNEP 2018), gender and ASGM (IISD 2022) and ASGM finance (planetGOLD Programme 2020).

## Conclusion

The data from recently submitted Minamata National Action Plans confirm that widespread use of mercury in ASGM is ongoing. Combating its use requires effective strategies that not only disseminate mercury-free technologies but do so in a way that responds to the geological, technical, social, and economic conditions in which ASGM is practiced. While this paper reviewed available research on these factors, much more publicly available information is needed to describe results, successes, challenges, failures, and lessons learned on interventions by multilateral and bilateral organizations and the private sector. Otherwise, new interventions are at risk of repeating the same mistakes. As work continues toward the goal of a mercury-free ASGM sector, more public exchange of field experience, through a technical and cultural lens, is needed to ensure effective uptake of alternative mercury-free technology.

## Disclaimer

The following disclaimer applies to all UNEP-affiliated authors, Ludovic Bernaudat, Kenneth J Davis, Malgorzata Stylo, Imelda Dossou Etui: The author alone is responsible for the views expressed in the publication and they do not necessarily represent the decisions or policies of the United Nations Environment Programme.

## Footnotes

1 Submitted NAPs can be found at https://www.mercuryconvention.org/en/parties/national-action-plans.

2 Dashboard is found at https://www.unep.org/globalmercurypartnership/insights-asgm-national-action-plans.

3 As used in the section, the term “mercury-free technology” means organization of knowledge for practical application. It encompasses the equipment (physical infrastructure) as well the processes, methods and activities involving the extraction and recovery of gold.
